# Interventional Radiology-Guided Foley Catheterization for Pelvic Fracture with Urethral Injury: A Single-Center Retrospective Case Series

**DOI:** 10.3390/jcm15135096

**Published:** 2026-06-30

**Authors:** Hong Chung, Min Ho Park, Euichul Jung, Sungyup Kim, Jun Gi Kim, Young Un Choi

**Affiliations:** 1 Department of Urology, Yonsei University Wonju College of Medicine, Wonju 26426, Republic of Korea; urohongchung@gmail.com; 2 Department of Radiology, Yonsei University Wonju College of Medicine, Wonju 26426, Republic of Korea; minnopak@naver.com (M.H.P.); marcy513@hanmail.net (E.J.); 3 Department of Surgery, Yonsei University Wonju College of Medicine, Wonju 26426, Republic of Korea; sykimvs@gmail.com; 4 Trauma Center, Wonju Severance Christian Hospital, Wonju 26426, Republic of Korea; witsumbe@yonsei.ac.kr

**Keywords:** pelvic fracture, urethral injury, foley catheterization, trauma

## Abstract

**Background/Objectives:** In male trauma patients suspected of having a pelvic fracture with urethral injury (PFUI), repeated urethral catheterization attempts can cause additional injury. Since 2021, our institution has been performing simultaneous percutaneous cystostomy using a guidewire and anterograde urethral Foley catheter insertion during pelvic angiography in patients with suspected PFUI. We aimed to analyze the characteristics and clinical course of this patient group. **Methods:** We retrospectively analyzed male trauma patients who were admitted to our emergency department between January 2021 and December 2025, and who underwent the aforementioned interventional procedure after standard Foley catheterization failed because of a pelvic fracture-associated urethral injury. The age, mechanism of injury, injury severity score (ISS), abbreviated injury scale, type of pelvic fracture, use of pelvic angiography, time from emergency room arrival to Foley catheterization, and administration of additional urological treatments were investigated. **Results:** Among 492 male patients with pelvic fractures, 11 underwent the procedure because of PFUI (age: 57.8 ± 13.9 years, ISS: 20.2 ± 9.6). Pelvic crushing was the most common injury mechanism, and pelvic angiography was performed in 81.8% of cases. The mean time from emergency room arrival to interventional Foley catheterization was 283 ± 250 min. Three patients required additional urological treatment after the acute phase, and all underwent endoscopic internal urethrotomy for urethral stricture. **Conclusions:** In cases in which hemodynamically unstable PFUI is suspected and initial urethral catheterization is difficult, Foley catheter insertion via interventional radiology may represent an alternative to conventional primary endoscopic realignment and suprapubic cystostomy.

## 1. Introduction

Traumatic genitourinary injury is found in up to 10% of patients with pelvic fractures. For patients with trauma, particularly severe trauma, Foley catheterization during initial treatment is a crucial and essential measure for evaluating and treating the patient’s condition. This procedure allows for the accurate measurement of hourly urine volume to check blood circulation and kidney function, which helps to determine whether fluid therapy or blood transfusion is being administered appropriately. In patients with lower abdominal trauma or pelvic fractures, Foley catheterization can be used to identify bladder or urethral injuries and diagnose hematuria. Additionally, if the patient is unconscious, requires bed rest, or is unable to urinate independently, Foley catheterization prevents bladder distension, facilitates urine drainage to comfort the patient and prevent bladder rupture. Furthermore, severe hematuria caused by urinary tract trauma prevents urethral obstruction by blood clots and enables continuous bladder irrigation [1].

However, in patients with trauma, there is a high probability of urethral injury if gross hematuria, blood at the urethral meatus, or a high-riding prostate is present. If urethral injury is suspected, retrograde urethrography should be performed first to diagnose the injury. However, it may be difficult to perform this test because of the characteristics of patients with trauma. In particular, injuries such as pelvic bone or straddle injuries may increase the suspicion of urethral injury [2]; in such cases, inappropriate Foley catheterization may worsen urethral injury. Therefore, it is safer to confirm the presence of urethral injury through retrograde urethrography, followed by Foley catheterization, if necessary. However, patients with pelvic fractures are often hemodynamically unstable and frequently experience concomitant injuries to other organs, which limit the ability to identify immediate urological injuries. Furthermore, if it is difficult to verify urination and measure urine volume, the administration of additional treatments may be challenging. In cases in which the patient is hemodynamically unstable and the urethra has not been properly evaluated, temporary cystostomy is recommended, followed by subsequent examination and treatment for urethral injury. However, progression of urethral injury without proper initial treatment can lead to complications such as urethral stricture and erectile dysfunction [3]. According to the literature, methods that can be attempted in such cases include early primary endoscopic realignment (PER), suprapubic cystostomy (SC), and subsequent surgical or endoscopic realignment [4]. However, there is still controversy regarding which of these two methods should be chosen because of their complications, and there is a lack of large-scale multicenter studies that can serve as a basis for this choice [4,5]. During the initial treatment of trauma patients, urological evaluation and treatment may be difficult because of multiple injuries, hemodynamic instability, or the inability to obtain early intervention from a urologist. Accordingly, since 2021, in cases where pelvic fracture with urethral injury (PFUI) is suspected and where Foley catheterization is not possible, radiologists at our institution have been performing emergency pelvic angiography in the early stages and simultaneously performing percutaneous cystostomy and (anterograde urethral) Foley catheterization via a guidewire. As a result, treatment is replaced with conservative observation alone when there are no early complications or when additional urological surgery is not required. In the current study, we examined the characteristics and prognoses of these patients.

## 2. Methods

### 2.1. Patients

In this retrospective study, we reviewed and analyzed the medical records of patients with pelvic bone fractures who were admitted to the emergency department of our institution between 1 January 2021, and 31 December 2025, and for whom Foley catheterization was impossible. This study was conducted in accordance with the Declaration of Helsinki and approved by the Institutional Review Board of Yonsei University Wonju College of Medicine (Approval No. CR326034). The requirement for informed patient consent for data use was waived because of the retrospective nature of the study and the use of de-identified data from medical records.

#### 2.1.1. Inclusion Criteria

The following patients were included in our study: male patients transferred to the emergency room with confirmed pelvic fractures who underwent radiological intervention because a urinary catheter could not be inserted properly owing to urethral injury; patients with detailed medical records; and patients admitted to the hospital with confirmed pelvic fractures via imaging examinations.

#### 2.1.2. Exclusion Criteria

The exclusion criteria were female patients, children aged < 18 years, pronounced dead upon emergency room arrival, and patients receiving management for direct genital or urethral injury other than pelvic fracture.

We included patients with trauma who were admitted with the diagnosis code “pelvic bone fracture” or “pelvic ring injury,” those with complete and detailed medical records, those whose pelvic injury was confirmed by radiology, and those in whom Foley catheterization was impossible at emergency room arrival. We excluded women, children aged < 18 years, patients who were pronounced dead upon arrival at the emergency room, and patients with only genital and urethral injuries without pelvic fractures.

**Figure 1:**
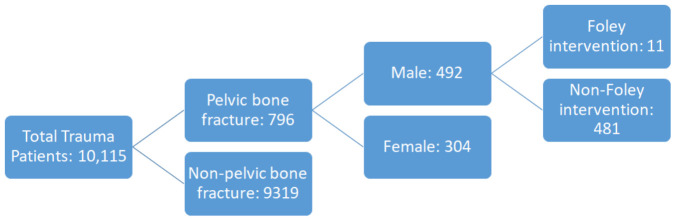
Flow chart of the included patients with pelvic bone fracture and urethral injury.

Among the 492 male patients with pelvic bone fractures, in whom conventional Foley catheterization was not possible due to urethral injury, 11 underwent Foley catheterization via interventional radiology (Figure 1).

### 2.2. Variables Analyzed

Age was recorded as a demographic variable. The clinical variables analyzed included the mechanism of injury (crush vs. non-crush), pelvic bone injury type (Young and Burgess classification), injury severity score (ISS), abbreviated injury scale score for each body region, presence or absence of pelvic angiography, and additional urological interventions. Pelvic angiography and urological intervention were performed by two interventional radiologists after the emergency room visit.

### 2.3. Statistical Analysis

For categorical variables, data were presented as frequencies and percentages, and the chi-square test was used for hypothesis testing. Fisher’s exact test was used when >20% of the cells in the contingency table had an expected count of <5, or when any cell had a frequency of zero.

For continuous variables, the normality of the data distribution was first tested using the Shapiro–Wilk test. Normally distributed variables were expressed as the mean ± standard deviation and were analyzed using an independent samples *t*-test. Non-normally distributed variables were expressed as medians and interquartile ranges and were analyzed using the Mann–Whitney *U* test.

Additionally, logistic regression analysis was performed to identify factors predicting PFIU patients. A stepwise variable selection method was used.

Statistical significance was defined as *p* < 0.05. All statistical analyses were performed using SAS version 9.4 (SAS Institute, Cary, NC, USA).

### 2.4. Procedure Method

**Figure 2:**
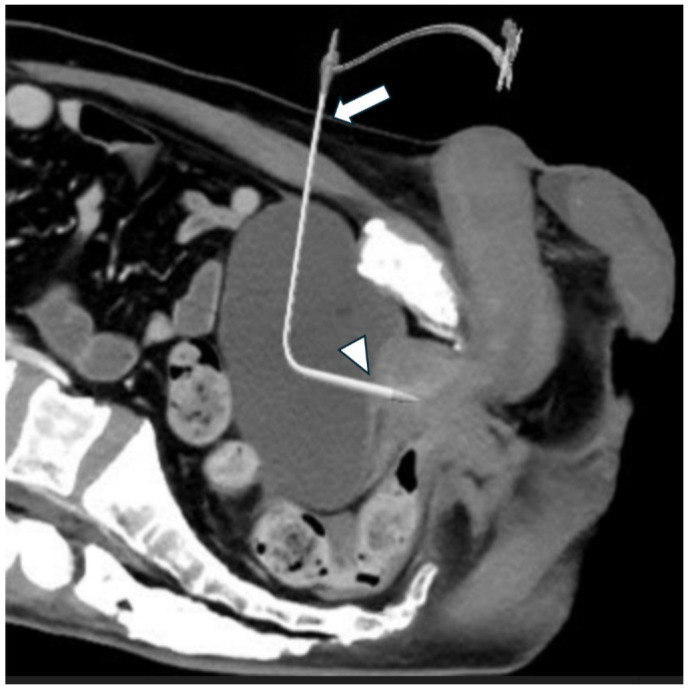
Interventional radiology procedure for Foley catheterization: Sagittal pelvic illustration showing suprapubic access. A 7-Fr, 25 cm sheath was inserted into the bladder (white arrow) with a manually angled (~90°) tip to facilitate the engagement of the internal urethral orifice (arrowhead).

The lower abdomen and perineum were sterilized and a retrograde approach through the external urethral meatus was initially attempted. A 5-Fr Kumpe catheter (Merit Medical, South Jordan, UT, USA) was used for contrast injection to delineate the urethral injury. If a 0.035-inch guidewire crossed the site of the injury, a Foley catheter was advanced over the wire; if unsuccessful, SC was performed under ultrasound guidance, and a 7-Fr, 25 cm sheath (Terumo, Tokyo, Japan) was inserted. The sheath tip was manually angled (~90°) to facilitate engagement of the internal urethral orifice (Figure 2).

**Figure 3:**
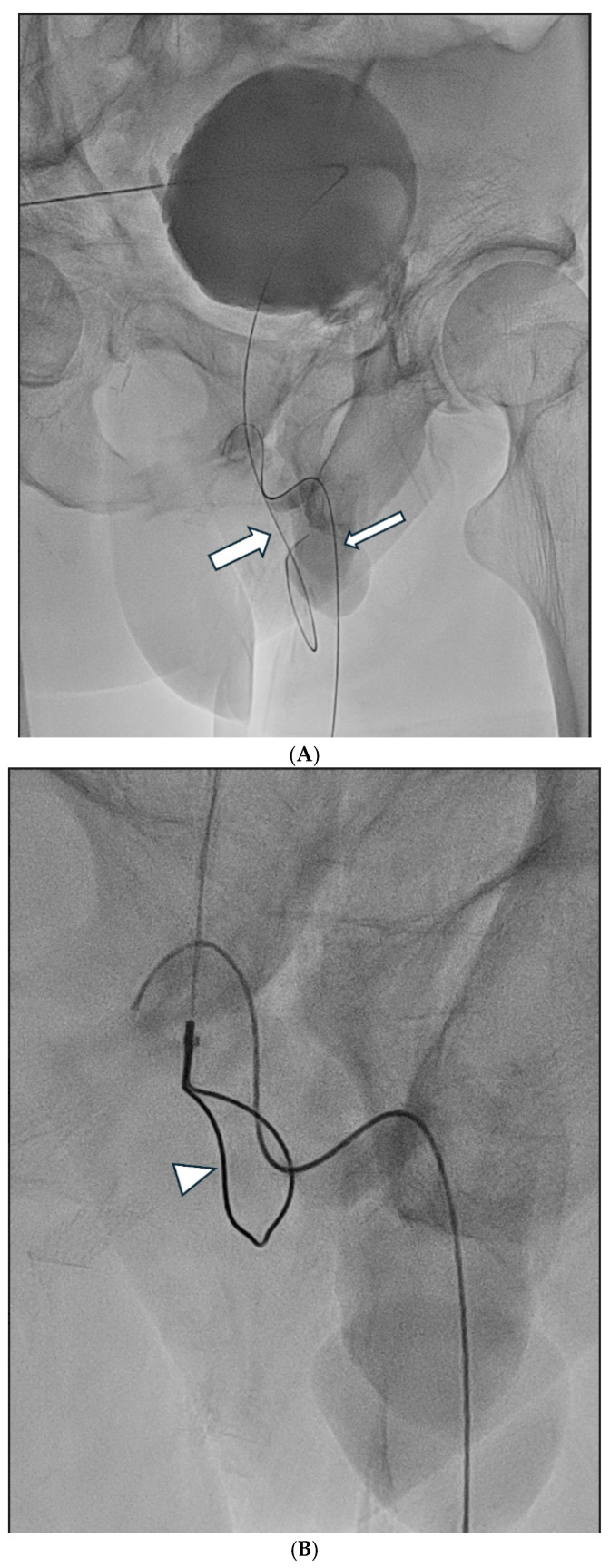

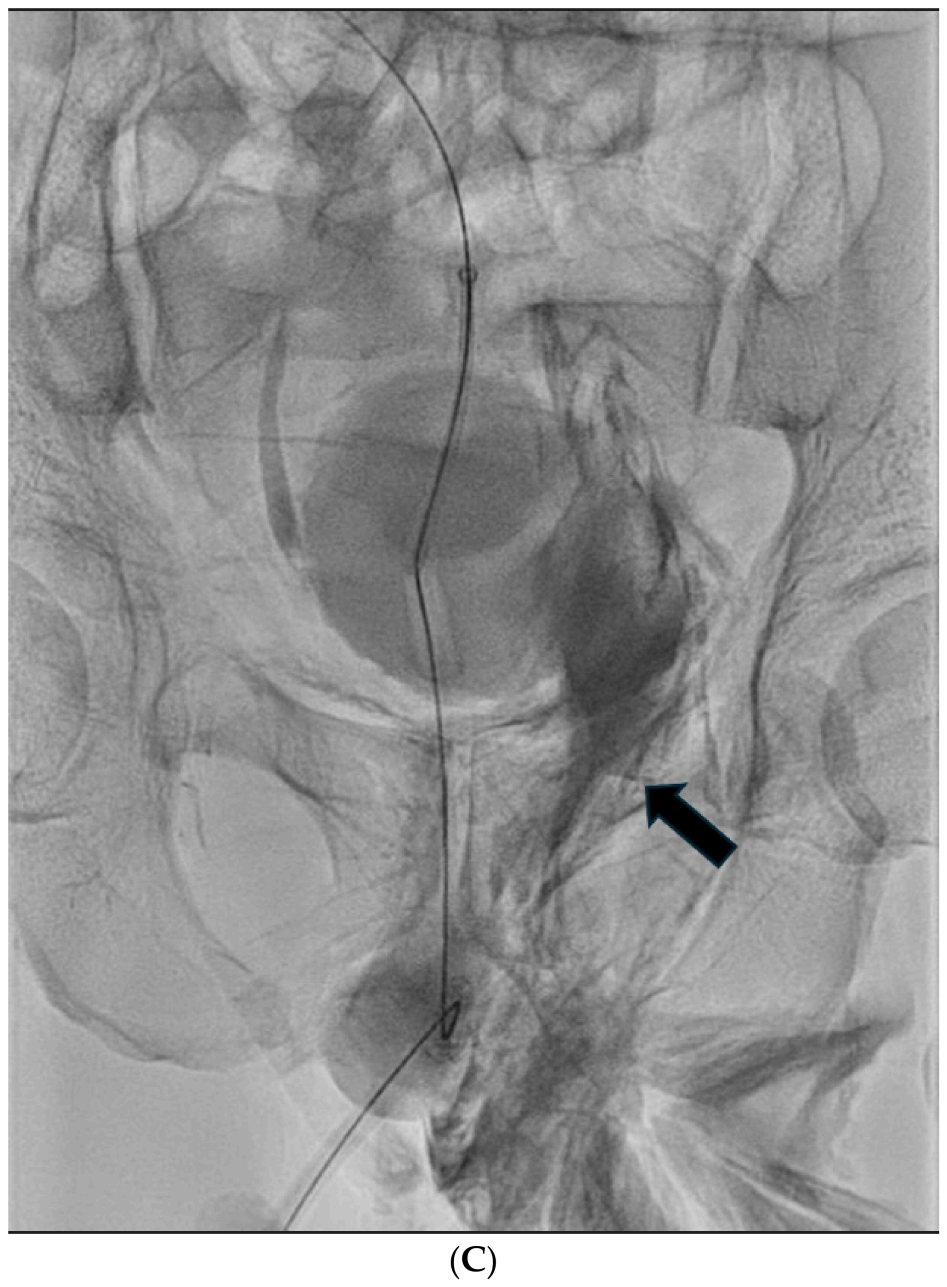
(**A**) Interventional radiology procedure for Foley catheterization: A 67-year-old man with traumatic membranous urethral disruption. During attempted Foley catheterization, fluoroscopy showed that antegrade (thick arrow) and retrograde (thin arrow) guidewires failed to traverse the disrupted segment. (**B**) Interventional radiology procedure for Foley catheterization: A 15 mm snare (arrowhead) was introduced via the suprapubic tract to capture the retrogradely advanced guidewire. (**C**) Interventional radiology procedure for Foley catheterization: The guidewire was successfully externalized, establishing through-and-through access for Foley catheter placement. Contrast extravasation was noted at the membranous urethral injury site (black arrow).

The guidewire was then advanced antegrade and externalized via the external urethral meatus, followed by Foley catheter placement. The sheath was removed, and a sterile compressive dressing was applied. In cases of complete disruption, the rendezvous technique was performed using an Amplatz Goose Neck Snare (10–20 mm; Medtronic, Minneapolis, MN, USA). The snare was introduced via the suprapubic tract and a guidewire advanced from the external urethral meatus was captured and externalized to establish through-and-through access. This procedure can also be performed in the opposite direction. Subsequently, the Foley catheter was advanced over the wire, the sheath was removed, and a sterile compressive dressing was applied (Figure 3).

**Figure 4:**
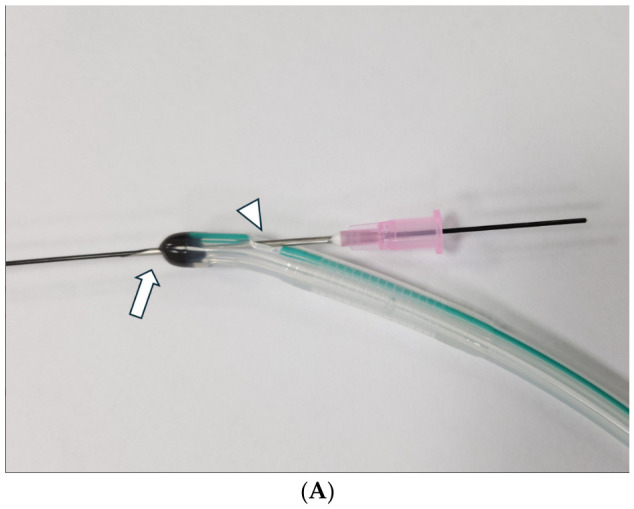

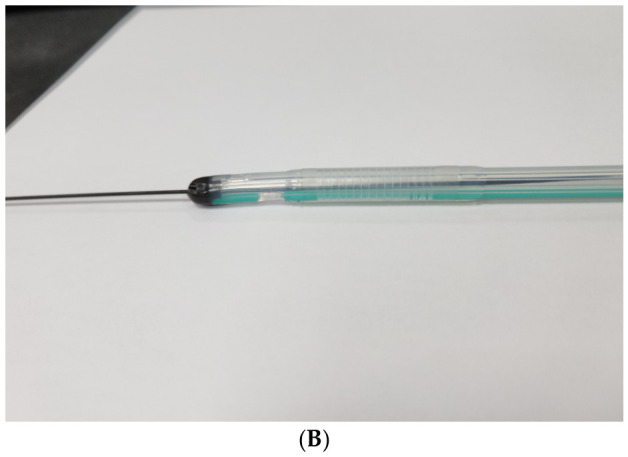
(**A**) To facilitate guidewire passage, the blind distal tip (white arrow) of the Foley catheter was punctured via the side hole (arrowhead) using an 18-gauge needle. (**B**) A 0.035-inch guidewire was advanced through the modified Foley catheter.

The size of the Foley catheter was selected at the operator’s discretion (typically 8–18 Fr). The Foley catheters used at our institution have a closed (blind) distal tip. To facilitate guidewire passage, the distal tip was punctured using an 18-gauge needle before insertion (Figure 4).

### 2.5. Urological Follow-Up

Catheter removal was performed within 2 to 3 months of insertion under the supervision of a urologist. Additionally, the final diagnosis of urethral stricture was confirmed at the urology outpatient clinic after 6 months; while most asymptomatic cases were monitored, cases with confirmed voiding dysfunction were verified via cystoscopy and urethrogram.

## 3. Results

### 3.1. Patient Information

During the study period, 10,115 trauma patients (6667 men and 3448 women) who visited the emergency room and were registered in the Korea Trauma Data Bank were identified. Among them, 796 (492 men and 304 women) had pelvic fractures. Eleven patients underwent Foley catheterization via interventional radiology for PFUI (Figure 1): eight patients had pelvic crush injuries, one patient had injuries from a motorcycle fall, and two patients had pedestrians-related pelvic impact injuries (Table 1).

### 3.2. General Demographics

The mean age of the included patients was 57.8 ± 13.9 years, and the mean ISS was 20.2 ± 9.6. Pelvic crush injury was the most common injury mechanism, accounting for 72.7% of cases. Pelvic angiography was performed in 81.8% of patients. The mean time from arrival at the emergency room to Foley catheterization was 283 ± 250 min. Additional urologic intervention after the acute phase was required in three patients (27.3%), all of whom underwent endoscopic internal urethrotomy for urethral stenosis (Table 2). All patients maintained normal urinary tract flow because of the successful procedure.

There were no statistically significant differences in age, ISS, injury mechanism, pelvic angiography findings, or time to Foley catheterization between the patients who underwent additional urological intervention and those who did not (all *p* > 0.05) (Table 3).

Foley catheter insertion was successfully achieved in all 11 patients through the above procedure, and no immediate complications such as bleeding or urinary extravasation, or urinary tract infection, were observed. The catheters were maintained for approximately 2–3 months and removed after consultation with a urologist; subsequently, urinary strictures were identified in 3 patients, requiring additional urological examinations and procedures (Table 4).

Comparing the crush and non-crush groups, no statistically significant difference was found in terms of additional urologic procedures. Foley catheterization tended to be performed faster in the crush group and showed differences depending on the mechanism; however, these differences were not significant. Fisher’s exact test showed no significant association between the injury mechanism and additional urological intervention (*p* = 1.000).

## 4. Discussion

The proportion of male patients with pelvic fractures for whom Foley catheter insertion could not be performed initially using conventional methods, as identified in this study, is similar to existing reports [6,7]. However, in the aforementioned study, cases involving minor injuries without difficult Foley catheterization were likely excluded. Therefore, the frequency appeared to be low because patients without difficult Foley catheterization could not be identified.

Notable aspects of this study include the high average age of the patients and the fact that they were severely injured, with an average ISS score > 20. Additionally, with the exception of two patients, all patients underwent angiography and embolization because of hemodynamic instability and radiological evidence of active pelvic bleeding. Furthermore, there were more patients with crush injuries were overwhelmingly more numerous than those with impact injuries. Foley catheterization was performed within 4 h, and only three patients required additional urological surgery or treatment. Regarding pelvic fracture types, three cases of anteroposterior compression (APC) type 2, four cases of lateral compression (LC) type 1, and four cases of LC type 2 were identified, all of which commonly showed ramus fractures. Most importantly, realignment was successfully achieved early via interventional radiology in all patients with PFUI who were unable to undergo Foley catheter insertion. Normal continence (voiding) was maintained in all patients.

In cases in which pelvic fractures and urethral injuries occur simultaneously, treatment primarily focuses on hemodynamic stabilization, correction of active bleeding sources, and coagulopathy. Therefore, in conventional methods, after forming an initial SC and after the patient’s condition stabilizes, the extent of urethral injury is assessed to determine the need for additional urological surgeries or procedures, or PER will be attempted from the start. However, disadvantages exist, such as the potential for future stenosis or erectile dysfunction, and the difficulty of endoscopic realignment in the early stages owing to poor visibility caused by bleeding. Furthermore, the method that should be prioritized remains controversial [8,9]. In the early 1990s, a Foley catheter was inserted using a guidewire 21 days after SC [10]. Since then, PER has been developed through advancements in endoscopy [11]. Although there have been discussions regarding the superiority of PER and SC, given the similar complication rates [12,13] and the lack of high-quality, large-scale data, it remains difficult to determine which is superior. Additionally, there are cases in which the damaged urethra heals naturally after conservative treatment following SC [14], but this is quite rare.

The radiological interventional procedures performed at our hospital offer several advantages.

First, when a patient requires angiography for a pelvic fracture, the radiology staff can perform the procedure in the radiology treatment room while simultaneously conducting Foley catheterization using a C-arm. Importantly, time and space are conserved as the same operator performs the procedure at the same location where the initial bleeding control is administered. Second, when a patient’s vital signs are initially unstable, it is difficult to accurately evaluate the urological damage. Furthermore, if only cystostomy is performed without realignment, problems such as stenosis, adhesions, or occlusion at the urethral injury site may arise, making management difficult. However, this method naturally induces tract healing and minimizes the extent of damage by avoiding invasive procedures, thereby preventing additional future interventions. Third, when urethral injury has progressed, the difficulty with inserting the Foley catheter and the resulting repeated insertion attempts can worsen the urethral injury; however, this can be minimized using the above method.

The follow-up results of the PER group treated via the urethra did not show a significant difference in the occurrence of complications compared with the group that underwent delayed surgery after SC. However, the rate of urethral stricture was 14% vs. 100%, indicating that endoscopy and management of adhesions were inevitably required in the SC group [15]. The PER group showed a significantly shortened spontaneous voiding time and reduced risk of urethral strictures [4]. A retrograde Foley catheter placement has several advantages. If endoscopic realignment is successful, an indwelling Foley catheter is possible via a guidewire; however, in cases of pelvic fracture, this procedure may promote further pelvic bone bleeding, making immediate treatment difficult. In addition, in the case of a high-riding bladder, the likelihood of immediate retrograde endoscopic realignment failure increases.

In contrast, immediate urethral realignment using a guidewire has the advantage of reducing patient discomfort and complications that may occur with a delayed operation after a successful procedure. It can be performed without changing the patient’s position because it requires only a guidewire. It also avoids visualization difficulties during the procedure, reduces unnecessary irritation, and minimizes extravasation caused by the irrigation fluid (saline) used during retrograde endoscopy. Although long-term and large-scale data on complications are required, in the current study, stenosis was the only complication in 3 of 11 cases. This was not significantly different when compared to the PER group, and considering that all cases were successful, the need for SC may be reduced [9,16,17].

Although pediatric patients were excluded from this study, it has been noted that, in this population, primary realignment is not superior to delayed urethroplasty in terms of the incidence of urethral stenosis, urinary incontinence, and erectile dysfunction [18]. However, given that this study did not use this method, future prospective studies and randomized controlled trials targeting pediatric patients with urethral injuries are warranted.

Straddle fractures (fracture of all four pubic rami), fractures of the inferior pubic branch with widened pubic symphysis, and Malgaigne fractures (double pelvic ring fracture dislocations) are known to be closely associated with urethral injury regardless of the presence or absence of sacroiliac joint distraction [19,20,21]. In the current study, three cases of APC type 2, four cases of LC type 1, and four cases of LC type 2 were identified as pelvic fractures, all of which had ramus fractures. The initial mechanism of PFUI was hypothesized to involve horizontal or shear forces acting through the membranous urethra at a site fixed to the genitourinary diaphragm [22,23,24]. However, owing to the recent rejection of the concept of the diaphragm, it has been suggested that this occurs because of dissection of the membranous and spherical urethra [25]. In patients with trauma, when a pelvic fracture is suspected during initial treatment, a portable pelvic anteroposterior radiograph obtained during an adjunctive primary survey can provide a rough estimate of the fracture; however, it is difficult to determine the exact type of fracture until hemodynamic stability is achieved and a computed tomography scan is performed. Therefore, regarding initial radiological pelvic fractures, the presence or absence of a ramus fracture is considered the most significant indication for suspected urethral injury.

At our institution, when performing percutaneous cystostomy and (antegrade urethral) Foley catheterization, the average time from arrival at the emergency room to catheter insertion was approximately 283 min. Considering that there was no significant difference in the insertion time among the patients and that the insertion time was not associated with additional urologic procedures related to strictures, it is thought that the procedure was performed at an appropriate time. Regarding the timing of removal after catheter insertion, endoscopic examination of the urethral rupture site 6 weeks after realignment showed that epithelialization was completed at 9 weeks in 83% of cases and at 12 weeks in 17% of cases. Therefore, the catheter should be maintained for at least 2 months; however, further large-scale studies are needed [26].

During the above period, there were a total of 11 such patients, all of whom underwent Foley catheterization using the above method; none of the patients underwent SC, and none of the patients died early after the above procedure.

This method is considered to be the optimal approach for inducing spontaneous healing in emergency trauma patients, without the need for additional invasive treatment by realigning the anatomical structure of the urethra. This method is believed to provide additional guidance for the treatment of urethral injuries in emergency trauma patients for whom a urologist cannot always be present. Nevertheless, this method requires that a radiologist be available for 24 h support and possesses the necessary experience and capability to perform the procedure. Furthermore, the small sample size may have diminished the significance of the patient characteristics and outcomes identified in this study. Therefore, a large-scale, multicenter, prospective, randomized study is required to obtain more accurate results. Additionally, a disadvantage of this study is that the extent of urethral injury could not be accurately assessed because the included patient group was unable to proceed with the initial treatment owing to multiple traumatic injuries and hemodynamic instability; thus, sufficient urological evaluation was not performed. Patients with minor injuries who did not exhibit bloody urine or those who did not experience issues with Foley catheterization were excluded from the study. Although it is likely that these patients had partial urethral injuries that would not encounter problems with conservative treatment, not all patients underwent urological evaluation.

## 5. Conclusions

Foley catheterization is essential in the initial treatment of patients with hemodynamic instability and pelvic fractures in whom urethral injury is suspected. However, if inserting a catheter through the urethra is difficult, percutaneous cystostomy and Foley catheter insertion may be considered. This approach enables the insertion of Foley catheters in pelvic fracture patients who cannot be palliatively inserted in the emergency room; it is expected to maintain normal urination and reduce potential complications such as urethral adhesions. However, more cases and follow-up examinations for more than six months are required.

## Figures and Tables

**Table 1:** Individual patient information.

No.	Age	Mechanism	ISS	AIS 1	AIS 2	AIS 3	AIS 4	AIS 5	Pelvic Bone Fracture Type	Pelvic Angiography	ER Visit to Foley Insertion (min)	Urologic Intervention	Urinary Continence
1	52	Crush injury	22	3	0	3	2	2	LC1	Yes	196	Yes	Yes
2	26	Crush injury	20	0	0	0	2	4	APC2	Yes	210	No	Yes
3	50	Non-crush	29	3	2	0	2	4	APC2	Yes	194	No	Yes
4	57	Non-crush	12	0	2	0	2	2	LC1	Yes	278	Yes	Yes
5	59	Crush injury	13	0	0	0	3	2	LC2	Yes	166	No	Yes
6	73	Crush injury	29	0	1	3	2	4	LC2	No	363	No	Yes
7	55	Crush injury	29	0	0	3	2	4	APC2	Yes	195	No	Yes
8	69	Non-crush	12	2	0	2	2	2	LC1	No	1045	No	Yes
9	68	Crush injury	9	0	1	0	2	2	LC1	Yes	184	No	Yes
10	66	Crush injury	38	0	1	3	2	5	LC2	Yes	97	Yes	Yes
11	61	Crush injury	9	0	0	0	2	2	LC2	Yes	185	No	Yes

ISS: Injury Severity Score; AIS: Abbreviated Injury Scale (AIS 1: Head and Neck, AIS 2: Face, AIS 3: Chest, AIS 4: Abdomen, AIS 5: Pelvic/Extremities); ER: Emergency room; LC: Lateral compression; APC: Anteroposterior compression.

**Table 2:** Patient baseline characteristics.

Variable	Value
Total number of patients	11
Age (years), mean ± SD	57.8 ± 13.9
**Mechanism of injury**	
Crush	8 (72.7%)
Non-crush	3 (27.3%)
**Pelvic bone fracture type**	
APC2	3 (27.3%)
LC1	4 (36.4%)
LC2	4 (36.4%)
**Pelvic angiography**	
Yes	9 (81.8%)
No	2 (18.2%)
Time to Foley insertion (min), mean ± SD	283 ± 250
**Additional urologic intervention**	
Yes	3 (27.3%)
No	8 (72.7%)
AIS 1 (Head and Neck), mean ± SD	0.73 ± 1.27
AIS 2 (Face), mean ± SD	0.64 ± 0.92
AIS 3 (Chest), mean ± SD	1.36 ± 1.43
AIS 4 (Abdomen), mean ± SD	2.09 ± 0.30
AIS 5 (Pelvic-extremities), mean ± SD	3.00 ± 1.10
ISS, mean ± SD	20.2 ± 9.6
**Urinary continence (yes)**	11 (100%)

ISS: Injury Severity Score, AIS: Abbreviated Injury Scale, APC: Anteroposterior compression, LC: Lateral compression, SD: Standard deviation.

**Table 3:** Comparison by additional urologic intervention.

Variable	Intervention (N= 3)	No Intervention (N = 8)	*p*-Value
Age (years)	58.3 ± 7.0	57.6 ± 15.6	>0.05
ISS	24.0 ± 13.0	18.8 ± 8.5	>0.05
Crush mechanism, n (%)	2 (66.7%)	6 (75.0%)	1.00
Time to Foley insertion (min)	190 ± 91	255 ± 275	>0.05
Pelvic angiography, yes, n (%)	3 (100%)	6 (75.0%)	>0.05

Mean ± SD, ISS: Injury Severity Score.

**Table 4:** Clinical Outcomes.

Variable	Value
Technical success	11 (100%)
Antegrade-only versus rendezvous technique	9 vs. 2
Immediate complications (yes)	
Bleeding	0/11
Infection	0/11
Catheter dislodgement	0/11
Urinary extravasation	0/11
Delayed structure	3/11 (27.27%)
Additional urologic intervention	3/11 (27.27%)

## Data Availability

The datasets used and/or analyzed in the current study are available from the corresponding author upon reasonable request.

## Abbreviations

The following abbreviations are used in this manuscript:

PFUI	Pelvic fracture with urethral injury
ISS	Injury severity score
AIS	Abbreviated injury scale
PER	Primary endoscopic realignment
SC	Suprapubic cystostomy
APC	Anteroposterior compression
LC	Lateral compression
SD	Standard deviation
